# Natural loss‐of‐function mutation of *EDR1* conferring resistance to tomato powdery mildew in *Arabidopsis thaliana* accession C24

**DOI:** 10.1111/mpp.12165

**Published:** 2014-07-09

**Authors:** Dongli Gao, Michela Appiano, Robin P. Huibers, Annelies E. H. M. Loonen, Richard G. F. Visser, Anne‐Marie A. Wolters, Yuling Bai

**Affiliations:** ^1^ Wageningen UR Plant Breeding Wageningen University Research Centre Droevendaalsesteeg 1 6708PB Wageningen the Netherlands; ^2^ Present address: Enza Zaden Beheer B.V. Haling 1E 1602 DB Enkhuizen the Netherlands

**Keywords:** EDR1, *Oidium neolycopersici*, resistance, tomato

## Abstract

To screen for potentially novel types of resistance to tomato powdery mildew *Oidium neolycopersici*, a disease assay was performed on 123 *Arabidopsis thaliana* accessions. Forty accessions were fully resistant, and one, C24, was analysed in detail. By quantitative trait locus (QTL) analysis of an F_2_ population derived from C24 × Sha (susceptible accession), two QTLs associated with resistance were identified in C24. Fine mapping of QTL‐1 on chromosome 1 delimited the region to an interval of 58 kb encompassing 15 candidate genes. One of these was *Enhanced Disease Resistance 1* (*EDR1*). Evaluation of the previously obtained *edr1* mutant of Arabidopsis accession Col‐0, which was identified because of its resistance to powdery mildew *Golovinomyces cichoracearum*, showed that it also displayed resistance to *O. neolycopersici*. Sequencing of *EDR1* in our C24 germplasm (referred to as C24‐W) revealed two missing nucleotides in the second exon of *EDR1* resulting in a premature stop codon. Remarkably, C24 obtained from other laboratories does not contain the *EDR1* mutation. To verify the identity of C24‐W, a DNA region containing a single nucleotide polymorphism (SNP) unique to C24 was sequenced showing that C24‐W contains the C24‐specific nucleotide. C24‐W showed enhanced resistance to *O. neolycopersici* compared with C24 not containing the *edr1* mutation. Furthermore, C24‐W displayed a dwarf phenotype, which was not associated with the mutation in *EDR1* and was not caused by the differential accumulation of pathogenesis‐related genes. In conclusion, we identified a natural *edr1* mutant in the background of C24.

## Introduction

Powdery mildews are able to colonize a wide variety of plant species, including Arabidopsis, and many economically important crops, such as wheat, barley and tomato. Resistance to powdery mildews can be manifested through the action of dominantly or semi‐dominantly inherited resistance genes (*R* genes). The most abundant dominant *R* genes encode proteins containing nucleotide‐binding site and leucine‐rich repeat (NBS‐LRR) domains, such as *Pm3b* in wheat (Yahiaoui *et al*., 2004), *MLA* alleles in barley (Seeholzer *et al*., 2010) and *Ol‐4* in tomato (Seifi *et al*., 2011). As a result of specific recognition of a matching pathogen‐encoded avirulence protein by the cognate *R*‐gene product, *R‐*gene‐mediated resistance is usually race or isolate‐specific (Ellis *et al*., 2000). In Arabidopsis, the only dominant *R* gene characterized to date, conferring resistance to powdery mildews, is *RPW8* (*Resistance to Powdery Mildew 8*), which is structurally different from common *R* genes and imparts resistance to several isolates of powdery mildew (Xiao *et al*., 2001).

Another form of powdery mildew resistance is governed by recessively inherited genes conferring race‐non‐specific resistance. Based on the resistance mechanism, they can be generally classified into three groups. Resistance in the first group is based on the loss of function of negative regulators of immune responses. An example is the *edr1* (*enhanced disease resistance 1*) mutation in Arabidopsis, resulting in resistance to powdery mildew *Golovinomyces cichoracearum* and the bacterial pathogen *Pseudomonas syringae* (Frye and Innes, 1998). *EDR1* encodes a putative mitogen‐activated protein kinase kinase kinase (MAPKKK), and is considered to be a negative regulator, because *edr1* resistance is caused by the activation of multiple defence responses, including increased defence gene expression and accelerated cell death response at the site of infection (Frye and Innes, 1998; Frye *et al*., 2001). Xiao *et al*. (2005) showed that *EDR1* negatively regulates *RPW8*. The resistance phenotype of *edr1* depends on the salicylic acid (SA) signalling pathway, because double mutants combining *edr1* with mutations that block SA defence responses or reduce SA production reverted to susceptibility for powdery mildew (Frye *et al*., 2001).

The second group is defined by loss of a host susceptibility factor required for pathogen growth. In a screen for Arabidopsis mutants showing resistance to powdery mildew *G. cichoracearum* independent of the constitutive expression of *PR1* (*Pathogenesis‐Related protein 1*) or the formation of lesions, the *pmr6* (*powdery mildew resistant 6*) mutant was identified (Vogel *et al*., 2002). *PMR6* encodes a putative pectate lyase and the loss‐of‐function mutation causes altered cell wall composition (Vogel *et al*., 2002). *pmr6‐*mediated resistance is independent of known defence responses, because mutations in genes encoding components of SA or jasmonate/ethylene pathways do not alter *pmr6* resistance status (Vogel *et al*., 2002). Furthermore, *pmr6* controls resistance to two powdery mildew species, but retains full susceptibility to unrelated pathogens, such as bacterium and oomycete species, suggesting that PMR6 may be a true powdery mildew compatibility factor (Micali *et al*., 2008; Vogel *et al*., 2002). Therefore, *pmr6* probably confers resistance as a result of a loss of a susceptibility factor rather than by activation of known host defence responses.

In the third group, well‐defined signalling pathways are not engaged, but resistance to unrelated pathogens is displayed. An example is *dmr1* (*downy mildew resistant 1*), which mediates resistance to both downy mildew *Hyaloperonospora arabidopsidis* and powdery mildew *Oidium neolycopersici* (*On*) (Huibers *et al*., 2013; Van Damme *et al*., 2009). *DMR1* encodes a homoserine kinase, and its impairment results in the accumulation of homoserine, which is responsible for the resistance to downy mildew (Van Damme *et al*., 2009). *dmr1*‐mediated resistance to downy mildew might trigger a novel defence pathway because the exogenous application of homoserine still induces resistance in the single mutant impaired in immune responses or double mutants combining *pmr4* (defective in the production of pathogen‐induced callose) with mutations that impair SA signalling pathways (Van Damme *et al*., 2009).

*On* is a powdery mildew species causing worldwide disease on tomato. Resistance genes have been identified in wild tomato species, including six monogenic genes comprising five dominant (*Ol‐1*, *Ol‐3*, *Ol‐4*, *Ol‐5*, *Ol‐6*) and one recessive (*ol‐2*) loci, and three polygenic resistance quantitative trait loci (QTLs) (Bai *et al*., 2003, 2005). However, to date, only the identities of *ol‐2*, *Ol‐4* and *Ol‐6* have been (partially) revealed (Bai *et al*., 2008; Seifi *et al*., 2011). *ol‐2* was shown to encode a non‐functional MLO protein which causes resistance as a result of enhanced cell death response and the deposition of a callose‐rich barrier (papilla) at the site of invasion. Hence, MLO is considered to be a negative regulator. *Ol‐4* and *Ol‐6* are probably *Mi‐1* homologues which encode NBS‐LRR‐type *R* proteins. They provide effective protection against three unrelated pests, i.e. powdery mildew, nematodes and aphids. However, neither gene has been cloned to date.

By studying 23 *Arabidopsis thaliana* accessions, Göllner *et al*. (2008) showed that *RPW8* (located on chromosome 3) and polygenic resistance are major sources of resistance to powdery mildew *G. orontii*. In the same study, seven of these accessions were challenged with *On.* Intriguingly, Sha, which contains *RPW8*, was resistant to three powdery mildew species, but susceptible to *On*, implying that *RPW8* is not effective against *On*. Furthermore, heterologous expression of *RPW8* genes in tomato did not result in resistance to *On* (Xiao *et al*., 2003). These data indicate that genetic factors for *On* resistance in Arabidopsis are different from those involved in resistance to *G. orontii*.

In this study, we employed the *On*–Arabidopsis pathosystem to: (i) determine the mode of inheritance of *On* resistance in natural accessions; and (ii) identify novel Arabidopsis genes conferring resistance to tomato powdery mildew. We were mainly interested in recessive genes, because these are less likely to be NBS‐LRR‐type *R* genes and may confer isolate‐non‐specific resistance. Ultimately, our goal is to silence or induce mutations in tomato orthologues of Arabidopsis resistance genes to achieve *On* resistance in tomato. Here, we describe the map‐based cloning of a recessive resistance locus in Arabidopsis, which turned out to be a natural mutation in the *EDR1* gene.

## Results

### Genetic analysis of *On* resistance in Arabidopsis accessions

To explore the natural variation for *On* resistance, 123 accessions (five plants per accession) were inoculated with *On* spores and evaluated on the basis of a disease index (DI) score ranging from ‘0’ (resistant) to ‘3’ (susceptible). In total, 40 accessions were fully resistant to *On* (DI = 0), whereas the others showed varying levels of susceptibility from low to high (Table S1, see Supporting Information). To determine the genetic mode of resistance, 19 resistant accessions were crossed with susceptible Col‐0 or Sha. The F_1_ plants (five plants per cross) from 18 crosses displayed a susceptible phenotype (DI > 0) (Table S2, see Supporting Information). To assess whether the resistance is mediated by a single gene or more than one gene, a *χ* ^2^ test was performed on respective F_2_ generations (Table S2). Segregation ratios (resistant : susceptible plants or resistant : intermediate : susceptible plants) following a single gene pattern were observed in four accessions. For the remaining 15 accessions, the segregation ratios were not compatible with a single‐gene hypothesis (*P* < 0.05), suggesting that resistance to *On* in Arabidopsis is mostly polygenic.

### Fine mapping of QTL‐1 controlling resistance to *On* in C24

**Figure 1:**
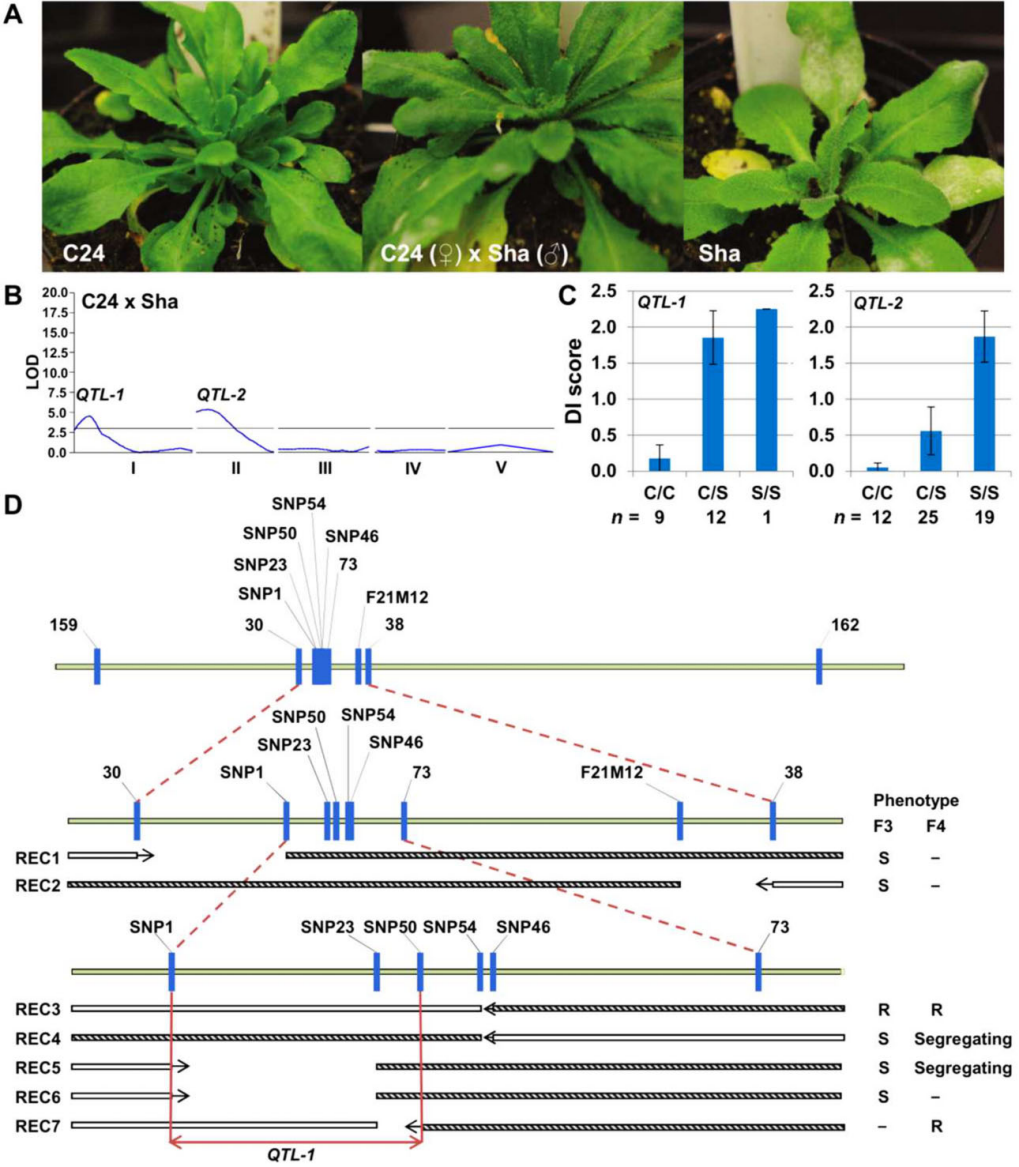
Quantitative trait locus (QTL) analysis of *Oidium neolycopersici* (*On*) resistance and fine mapping of QTL‐1 on chromosome 1 in C24 × Sha. (A) Symptoms of *On* infection on C24, F_1_ C24 × Sha and Sha. (B) Two QTLs located on chromosomes 1 and 2 were identified. LOD, logarithm of odds. (C) Disease index (DI) after *On* infection. Class C/C is homozygous for the C24 allele, C/S is heterozygous and S/S is homozygous for the Sha allele. For each class, the DI value is the average score of F_2_ plants with the designated genotype for marker 159 linked to QTL‐1 on chromosome 1, or for marker 515 linked to QTL‐2 on chromosome 2. (D) Markers used for the fine mapping of QTL‐1 (Table S4) are indicated. The distance between markers is proportional to the physical distance. White bars represent regions homozygous for the C24 allele, and shaded bars represent heterozygous regions. The space in between white and shaded bars denotes a crossover event between two flanking markers for each recombinant. The arrows point towards the interval in which QTL‐1 resides. For each recombinant (REC), the phenotype of F_3_ and/or F_4_ populations is indicated. S, susceptible; SNP, single nucleotide polymorphism; R, resistant.

C24 is one of the accessions exhibiting absolute resistance. It was crossed with susceptible accession Sha to generate a mapping population. The F_1_ plants were susceptible (Fig. 1A), and the segregation ratio of F_2_ plants suggested the involvement of more than one resistance gene (Table S2). Preliminary QTL analysis of 96 F_2_ plants with 21 indel markers (Table S3, see Supporting Information) covering all five chromosomes resulted in the identification of two QTLs with a logarithm of odds (LOD) score higher than 2.5 (Fig. 1B). QTL‐1 was located on chromosome 1 and acted in a recessive manner, as only plants homozygous for the C24 allele in this region were resistant (Fig. 1C). QTL‐2 on chromosome 2 acted in a semi‐dominant manner (Fig. 1C). To separate the effects of the two QTLs, we selected single F_2_ plants showing a heterozygous genotype at one QTL locus and homozygous for the Sha allele at the other locus, and selfed these to produce F_3_ progeny. The analysis of F_3_ progeny showed a tight correlation between phenotype and genotype for QTL‐1 (Fig. S1A, see Supporting Information); plants (*n* = 37) with the C24 genotype were fully resistant or only slightly infected (with a mean DI below 0.5), whereas plants with a heterozygous (*n* = 25) or Sha (*n* = 1) genotype supported a high level of fungal sporulation (with a mean DI in the range 1–3). Surprisingly, only one plant had the Sha genotype at QTL‐1, showing a skewed ratio of genotypes. For QTL‐2, plants with the C24 genotype and heterozygous plants showed full resistance, except for two heterozygous plants (Fig. S1B). The DI for plants with the Sha genotype was in the range 0–2 (Fig. S1B). Considering that QTL‐1 confers full resistance and shows a tight correlation between genotype and phenotype, we focused our attention on the cloning of QTL‐1.

To fine map QTL‐1, two flanking markers, 159 and 162 (Table S4, see Supporting Information), were used to screen 136 F_3_ plants derived from a single F_2_ plant which is heterozygous at the QTL‐1 locus and homozygous for the Sha allele at the QTL‐2 locus. Two recombinants REC1 and REC2 were obtained (Fig. 1D), and examination of their responses to *On* narrowed down the QTL‐1 region between markers 30 and 38. Recombinants were sought between these two markers by the analysis of 3552 F_3_ plants. Five informative recombinants (REC3–REC7) were obtained (Fig. 1D), and disease assays were performed using F_3_ recombinants or their F_4_ progenies, or both. By combining the genotypic and phenotypic data, the QTL‐1 interval was reduced to a 58‐kb chromosomal region between markers single nucleotide polymorphism 1 (SNP1) and SNP50 (chromosome 1 nucleotides 2 754 401–2 811 983; Fig. 1D).

### A natural mutation in *EDR1* confers resistance to *On*

**Figure 2:**
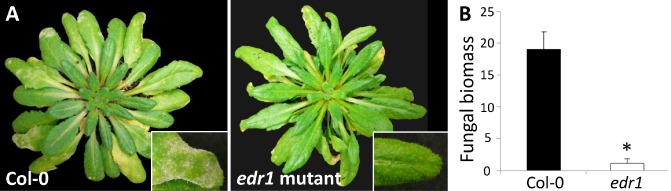
The *edr1* (*enhanced disease resistance 1*) mutation confers resistance to tomato powdery mildew O *idium neolycopersici*. (A) Fungal growth on Col‐0 plant and *edr1* mutant. (B) Fungal biomass quantification. Values were normalized relative to *act2*, and calibrated to levels on *edr1* mutants. Error bars represent standard deviation of three biological replicates and, for each replicate, rosette leaves were collected. Asterisk indicates significant difference from the control according to independent‐samples *t*‐test: *P < 0.05. A representative of two experiments is presented.

The 58‐kb interval between markers SNP1 and SNP50 encompasses 15 candidate genes. Interestingly, one of these is *EDR1* (At1g08720). Previously, a mutated *edr1* allele was obtained by γ‐irradiation in the background of Col‐0 (Frye and Innes, 1998). The induced mutation caused a premature stop codon in the fourth exon of EDR1. The *edr1* mutant was shown to be resistant to powdery mildew *G. cichoracearum*. To investigate whether *EDR1* is a good candidate for QTL‐1, we challenged the *edr1* mutant from Frye and Innes (1998) with tomato powdery mildew *On*. Col‐0 showed clear symptoms of infection by *On*, whereas *edr1* was free of symptoms (Fig. 2A). Quantification of fungal DNA indicated an approximately 20‐fold decrease in fungal biomass on the *edr1* mutant compared with Col‐0 (Fig. 2B).

**Figure 3:**
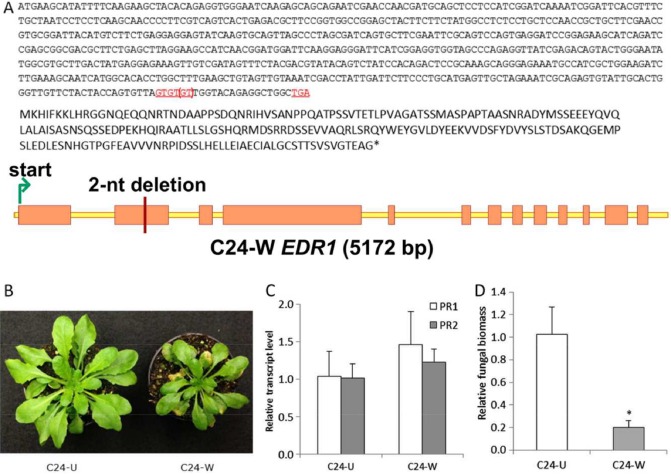
Characterization of C24‐W. (A) Mutation in the *EDR1* (*Enhanced Disease Resistance 1*) gene of C24‐W. Top panel shows that, among three GT repeats (red, underlined), one repeat was missing in C24‐W, leading to the occurrence of a premature stop codon TGA (red, underlined). The middle panel shows the resulting protein sequence when one GT repeat is missing. The bottom panel depicts the position of the two‐nucleotide (nt) deletion in *EDR1* of C24‐W. (B) Size differences of C24‐U and C24‐W plants. (C) Relative level of expression of defence genes *PR1* (*Pathogenesis‐Related gene 1*) and *PR2* (*Pathogenesis‐Related gene 2*) in plants not inoculated with the pathogen. (D) Fungal biomass quantification in C24‐W compared with C24‐U. Values were normalized relative to *act2*, and calibrated to levels in C24‐U plants. Error bars represent standard deviation of three biological replicates and, for each replicate, rosette leaves were collected. Asterisks indicate significant difference from the control according to independent‐samples *t*‐test: *P < 0.05. A representative of two experiments is presented.

These results showed that *EDR1* is a good candidate for QTL‐1. Examination of the protein sequences of *EDR1* from C24 (accession no. EF470629) and Col‐0 (accession no. AF305913) in the National Center for Biotechnology Information (NCBI) database revealed an amino acid difference (V395E) in the fourth exon in a non‐conserved region of the protein. To investigate whether this difference is associated with resistance, we sequenced the coding regions of *EDR1* in parental lines C24 and Sha. Surprisingly, a premature stop codon in the second exon of *EDR1* was produced in C24 as a result of the loss of two nucleotides (GT) compared with the Sha allele (Fig. 3A, genomic sequence chromosome 1, position 2 775 090–2 775 091). *EDR1* is located between markers SNP1 and SNP23 (Fig. 1C). Recombinants REC3 and REC7 were homozygous for the C24 allele of both markers, and they were both resistant to *On*. Therefore, we expected them to contain the *edr1* mutation. Sequencing results confirmed that REC3 and REC7 indeed carried the *edr1* mutation (data not shown), indicating that *edr1* is correlated with resistance to *On*. Using an F_4_ population derived from an F_3_ plant heterozygous for QTL‐1 and homozygous for Sha alleles at QTL‐2, the association between the *edr1* mutation and resistance was further confirmed by phenotyping (a disease test) and genotyping with a CAPS marker that can distinguish the C24(‐W) allele from the Sha allele of *EDR1* (Fig. S2, see Supporting Information). Remarkably, as observed previously (Fig. S1A), a skewed segregation of genotypes was again obtained. From 96 F_4_ plants, 31 were homozygous for the C24‐W *edr1* allele, 63 were heterozygous and only two were homozygous for the Sha *EDR1* allele.

Because the deletion of dinucleotide GT (nucleotides 1033–1034) was not present in EDR1 (accession no. EF470629) of C24, we suspected that our C24 germplasm (referred to as C24‐W) might be different from other C24 sources. Therefore, part of the second exon of *EDR1* was sequenced from plants of the stock (C24‐stock) from which we obtained C24‐W, and two other C24 sources (referred to as C24‐H and C24‐U, see Experimental procedures). Sequencing results showed that none of these C24 sources carried the mutation (Fig. S3, see Supporting Information). One possibility is that the accession we used was not C24. To exclude this possibility, we used 12 indel markers from all five chromosomes of Arabidopsis (Table S5, see Supporting Information) to genotype C24‐W, C24‐H, C24‐U, C24‐stock and Col‐0, as well as the DNA of the parental plants C24 and Sha used for crossing and for the development of all the markers for mapping in the F_2_ population. The resulting genotyping data showed that all C24 sources had the same marker pattern (Fig. S4, see Supporting Information), indicating that C24‐W is not different from the other sources of C24. As these data may not be conclusive, we searched for a C24‐specific DNA signature, a SNP of MIR164A unique to C24 (the C on chromosome 2 at position 19 520 846 is substituted by T in C24), which was found by analysing 96 Arabidopsis accessions (Todesco *et al*., 2012). polymerase chain reaction (PCR) products containing the MIR164A SNP were obtained from the genomic DNA of several sources of C24 and also Col‐0. Sequencing results showed that all C24 sources, including C24‐W, carried T instead of C at this position, whereas Col‐0 contained the expected T (Fig. S5, see Supporting Information). Thus, we confirmed that C24‐W is truly of C24 lineage.

We chose C24‐W and C24‐U for further analysis. A notable difference was that C24‐W plants were smaller than those of C24‐U (Fig. 3B). A reduced plant size is observed in a number of Arabidopsis mutants which constitutively accumulate high levels of SA (Lu *et al*., 2003). As an elevated level of SA induces the expression of *PR* genes, the basal expression levels of *PR1* and *PR2* in non‐inoculated plants of C24‐W and C24‐U were compared. The results showed that transcript levels of *PR1* and *PR2* were not increased significantly in C24‐W compared with C24‐U (Fig. 3C), suggesting that the smaller size of C24‐W is not caused by the accumulation of SA. As C24‐U does not carry the *edr1* mutation, it was expected to be less resistant than C24‐W to *On*, which was confirmed by quantification of the fungal biomass (Fig. 3D).

### Induction of PR gene expression and cell death are associated with the resistance conferred by the C24‐W *edr1* mutation

**Figure 4:**
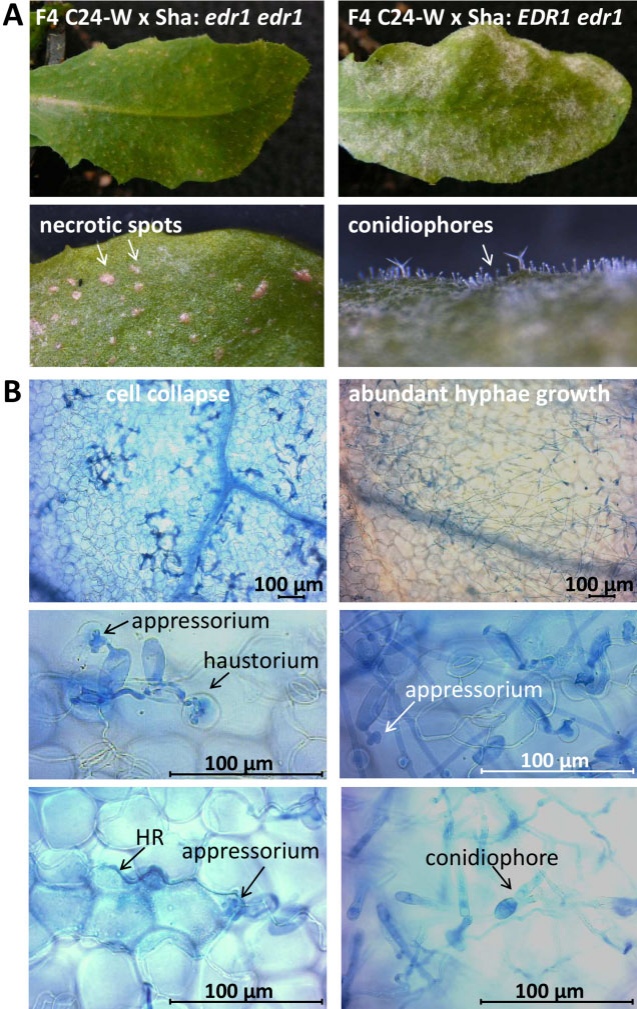
Macroscopic and microscopic images of F_4_ plants homozygous for the C24‐W *edr1* (*enhanced disease resistance 1*) allele (*edr1/edr1*), or heterozygous (*EDR1/edr1*), at 8 days post‐inoculation with *Oidium neolycopersici* (*On*). Left panel: F_4_ plants homozygous for the C24‐W *edr1* allele; right panel: heterozygous F_4_ plants. (A) Macroscopic symptoms after *On* inoculation. (B) Images of histological study showing fungal growth and cell death. Compared with heterozygous F_4_ plants, fungal growth was greatly restricted after haustorium formation on the resistant *edr1edr1* F_4_ plants, and cell death was observed in many epidermal cells intruded by fungal haustoria. HR, Hypersensitive Response.

On pathogen infection, the *edr1* mutant derived from Col‐0 (Frye and Innes, 1998) showed accelerated cell death and elevated *PR1* expression, although the mutant did not express PR1 constitutively. In an attempt to study the resistance mechanism conferred by the *edr1* mutation in C24‐W, a histological analysis was performed on *On*‐inoculated leaves of F_4_ plants homozygous for the C24‐W *edr1* allele and on heterozygous *EDR1edr1* F_4_ plants. Samples were taken 3, 6 and 8 days post‐inoculation (dpi). Macroscopically, no fungal sporulation was observed on F_4_ plants homozygous for the mutant allele (*edr1edr1*) (Fig. 4A) relative to heterozygous F_4_ plants. Instead of fungal colonies, necrotic spots appeared on inoculated leaves of *edr1edr1* plants (Fig. 4A). Microscopically, fungal growth was greatly restricted after haustorium formation on the resistant F_4_ plants starting from 6 dpi (Fig. 4B). Cell death was observed in many epidermal cells intruded by fungal haustoria (Fig. 4B), suggesting that the resistance conferred by the C24‐W *edr1* allele is post‐haustorial.

**Figure 5:**
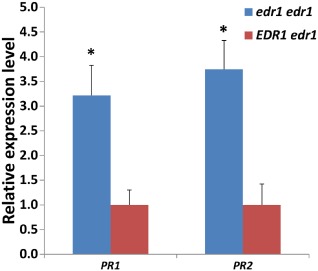
Relative *PR* (*Pathogenesis‐Related*) gene expression levels in F_4_ plants homozygous for the C24‐W *edr1* allele (*edr1edr1*), or heterozygous (*EDR1edr1*), at 6 days post‐inoculation with *Oidium neolycopersici*.

Next, we investigated *PR1* and *PR2* gene expression levels in homozygous *edr1edr1* F_4_ plants at 6 dpi. A significant increase in expression level (more than three‐fold) of both *PR* genes was observed in the F_4_ plants homozygous for the C24‐W *edr1* allele compared with the heterozygous F_4_ plants (Fig. 5). This suggests the involvement of the SA pathway in the resistance to *On* conferred by the C24‐W *edr1* allele.

### Suppression of putative homologues of *EDR1* in tomato

**Figure 6:**
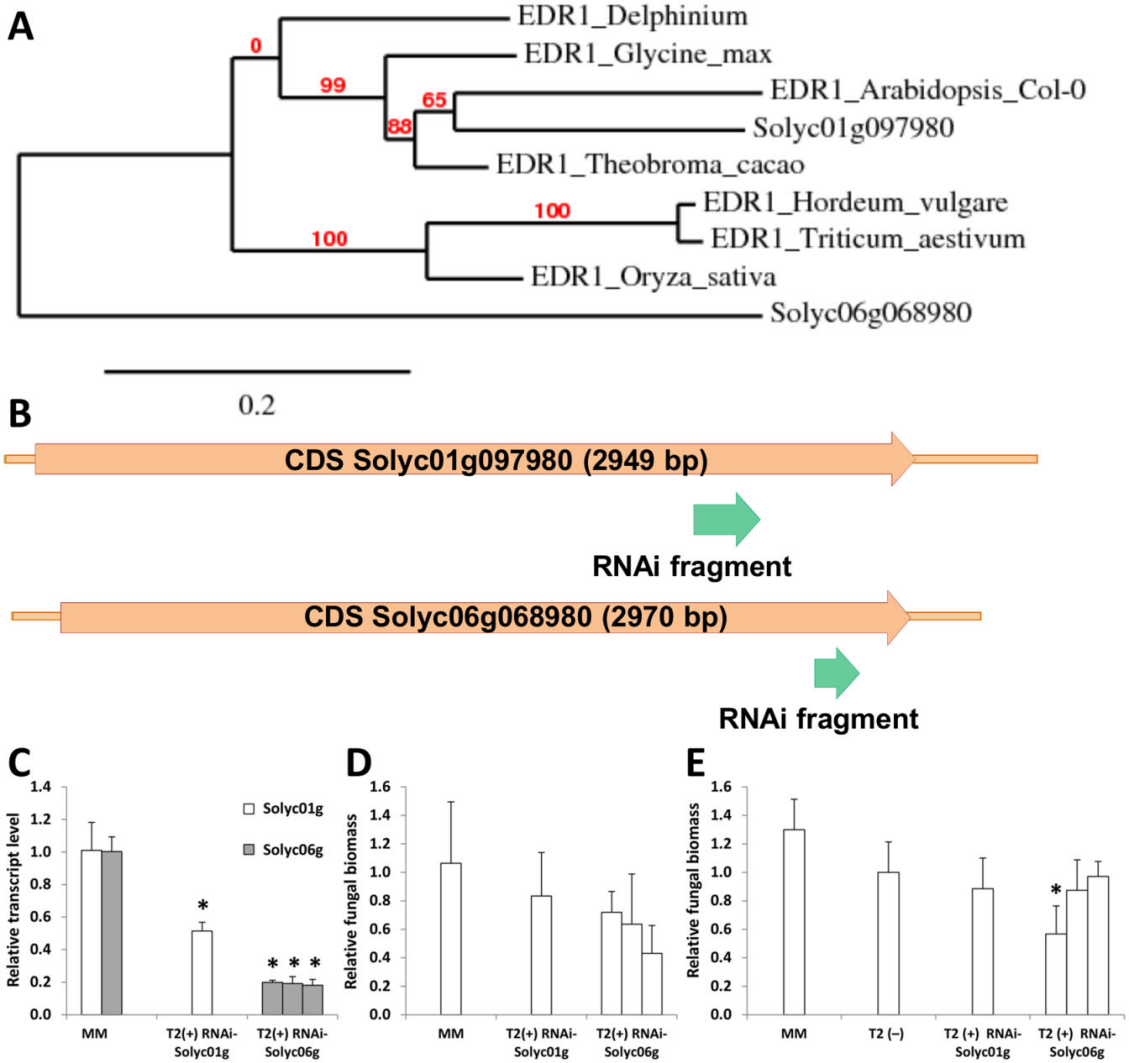
Suppression of putative homologues of *EDR1* (*Enhanced Disease Resistance 1*) in tomato did not affect the susceptibility level of cultivar Moneymaker (MM). (A) Phylogenetic analysis of protein sequences of tomato *EDR1* candidates compared with EDR1 from Arabidopsis and other plant species. (B) Representation of fragments from the coding sequences of the tomato *EDR1* candidates used in RNAi constructs. (C) Relative expression of *Solyc01g097980* (*Solyc01g*) and *Solyc06g068980* (*Solyc06g*) in silenced lines. (D) Relative fungal growth in T2 plants containing the silencing construct [T2(+)] compared with MM (results of the first experiment). (E) Relative fungal growth in T2 plants containing the silencing construct [T2(+)] compared with T2 plants without the silencing construct [T2(–)] (results of the second experiment). Values were normalized relative to *Elongation Factor 1α* (EF), calibrated to levels in untransformed MM plants or non‐transgenic T2 plants. Error bars represent standard deviation of at least three biological replicates and, for each replicate, third and fourth leaves were pooled. Asterisks indicate significant difference from the control according to one‐way analysis of variance or *t*‐test: *P < 0.05.

The aim of our study was to identify genes conferring broad‐spectrum resistance to powdery mildews in Arabidopsis, and subsequently to investigate whether putative orthologous genes in tomato also confer resistance to powdery mildew. With the protein sequence of Arabidopsis EDR1 as a query, multiple genes showing a relatively high level of homology were found in the tomato genome database SGN (Sol Genomics Network). We chose the first two genes, *Solyc01g097980* (*Solyc01g*) and *Solyc06g068980* (*Solyc06g*), to investigate their involvement in resistance. Phylogenetic analysis of these two tomato genes with the protein sequences of Arabidopsis EDR1 and EDR1 sequences from other species indicated that *Solyc01g* is much more closely related than *Solyc06g* to EDR1 from Arabidopsis and other species (Fig. 6A). The protein sequences encoded by *Solyc01g* and *Solyc06g* show 56% and 45% identity with the Arabidopsis EDR1 protein, respectively, whereas they show 42% identity with each other. The protein encoded by *Solyc01g* (accession no. AJ005077) probably is an EDR1‐like MAPKKK protein, because it is more similar to Arabidopsis EDR1 protein than to any of the five Arabidopsis EDR1 paralogues (Frye *et al*., 2001). Furthermore, the kinase domains of the *Solyc01g*‐encoded protein and Arabidopsis EDR1 show 86% identity (Frye *et al*., 2001). Tomato cultivar Moneymaker (MM) was transformed with RNAi silencing constructs (Fig. 6B), and several primary transformants (RNAi‐*Solyc01g* and RNAi‐*Solyc06g*) were obtained. These were selfed to produce T2 progeny. One T2 family for *Solyc01g* and three for *Solyc06g* were obtained. Nine plants harbouring the *NPTII* (*Neomycin Phosphotransferase II*) resistance gene from each T2 family were challenged with *On*. All supported abundant powdery mildew sporulation, comparable with the untransformed control, as judged by visual inspection. Subsequently, three plants from each T2 family were analysed for the expression of the targeted *EDR1* homologues, and the fungal biomass was quantified. Although significantly reduced expression of *Solyc01g* and *Solyc06g* was detected in both the RNAi‐*Solyc01g* and RNAi‐*Solyc06g* lines, respectively (Fig. 6C), the level of fungal growth in the transgenic T2 plants was only slightly decreased relative to the level in MM (Fig. 6D). We repeated the disease assay with more plants per T2 family, and included non‐transgenic T2 plants for fungal biomass quantification (at 15 dpi). In this experiment, we observed that all T2 plants (transgenic and non‐transgenic) showed a reduced fungal biomass relative to MM (Fig. 6E). The transgenic T2 plants T2(+) did not show a significant reduction in fungal biomass compared with the non‐transgenic T2 plants T2(–), except for one T2 family in which *Solyc06g* was silenced. This suggests that the silencing of *Solyc01g* and *Solyc06g* separately did not result in resistance against tomato powdery mildew.

## Discussion

The reference species *A. thaliana* displays abundant genetic variation among wild accessions (Alonso‐Blanco and Koornneef, 2000), which was illustrated by our results obtained after challenging 123 accessions with virulent tomato powdery mildew *On*. With the inoculum dosage routinely used, 40 accessions showed complete resistance (Table S1). Segregation analysis of 19 crosses in F_1_ and F_2_ (Table S2) indicated that polygenic resistance to *On* is more common than monogenic resistance. This observation was also made in a study of resistance to powdery mildew in multiple Arabidopsis accessions (Göllner *et al*., 2008). Both observations support the notion that polygenic resistance seems to be more common in interactions of powdery mildews with Arabidopsis than with barley, and also over‐represented relative to other Arabidopsis plant–pathogen interactions (Schulze‐Lefert and Vogel, 2000).

In this study, we identified a natural mutation of *EDR1* in the C24 background, resulting from the deletion of two nucleotides from a dinucleotide repeat array (GT)_3_. Eukaryotic genomes contain strings of DNA in which a single base or a small number of bases are repeated (microsatellites). Rearrangement can occur within repeated sequences, resulting in repeat addition and deletion (Flavell, 1986). This is probably caused by slippage during DNA replication (Ellegren, 2004). AC/GT repeats are scarce in plants in comparison with observations in mammalian genomes (Lagercrantz *et al*., 1993). Examination of dinucleotide repeats in Arabidopsis confirmed that AC/GT is least abundant (Marriage *et al*., 2009; Morgante *et al*., 2002). Marriage *et al*. (2009) estimated the mutation rate of dinucleotide repeats in Arabidopsis, and revealed that the majority of mutations are gains or losses of a single repeat, where the AC/GT motif is the least mutable. The mutation rate is positively affected by repeat length across motifs, but the AC/GT motif does not fit this general trend. Although meiotic and mitotic errors could not be distinguished in the study, they suggested that meiotic errors are more likely to contribute to the mutation rate. Our observation that C24‐W contains a dinucleotide deletion from a microsatellite sequence, whereas all other sources of C24 do not contain this mutation, suggests that the mutation is a recent event.

It is notable that C24‐W carrying the *edr1* mutation exhibits reduced stature, whereas the *edr1* mutant in the Col‐0 background does not. Reduced stature of C24‐W is not caused by the differential expression of *PR* genes (Fig. 3C), nor is it associated with the *edr1* mutation, because progenies only segregating for QTL‐1 did not show dwarfing. In contrast, progenies only segregating for QTL‐2 showed size differences. Almost all plants homozygous for the C24 alleles of markers linked to QTL‐2 showed reduced stature (Table S6, see Supporting Information). However, the QTL‐2 region has not been fine mapped to date, and this prevents us from unravelling the mechanism underlying the dwarf phenotype.

Another remarkable observation was the skewed ratio of genotypes in both F_3_ and F_4_ populations in which only the QTL‐1 allele is segregating. There was a shortage of plants homozygous for the Sha allele (the *EDR1* allele) (Figs S1 and S2). Possibly, plants homozygous for the Sha allele in the QTL‐1 region germinate more slowly than plants homozygous and heterozygous for the C24 allele. In our experiments, we sowed seeds together in one pot and, after germination, transplanted the small seedlings to individual pots. This may have resulted in the selection of seedlings only from the fast germinating seeds.

Accession C24 shows broad‐spectrum resistance to several unrelated pathogen species. C24 exhibits resistance to three species of powdery mildew, i.e. *G. orontii*, *G. cichoracearum* and *G. cruciferarum* (Göllner *et al*., 2008). Mapping of the gene(s) underlying this resistance has been unsuccessful (Göllner *et al*., 2008). In addition, C24 provides downy mildew isolate‐specific resistance and dominant resistance against the bacterium *Pseudomonas syringae* pv. *tomato* DC3000 (Lapin *et al*., 2012). Furthermore, C24 confers effective resistance against *Cucumber mosaic virus* mediated by a coiled coil (CC)‐NBS‐LRR‐type protein RCY1 (Takahashi *et al*., 2002). Therefore, C24 seems to be an example of the natural pyramiding of different resistance loci.

Here, we showed that C24 without the *edr1* mutation was less resistant to tomato powdery mildew *On* than C24‐W carrying the mutation (Fig. 3D), but, compared with Col‐0, it was less susceptible, as judged by visual inspection and after fungal biomass quantification (Fig. S6, see Supporting Information). This might be explained by elevated levels of SA, hydrogen peroxide and the expression of SA‐mediated defence‐related genes, such as *PR1*, in C24 (Bechtold *et al*., 2010; Lisec *et al*., 2008). However, these inherent traits do not necessarily contribute to pathogen resistance, because Col‐0 introgression lines containing resistance QTLs to downy mildew *H. arabidopsidis* did not show enhanced expression of *PR1* compared with susceptible Col‐0 (Lapin *et al*., 2012).

The paradigm examples of naturally occurring loss‐of‐function mutations conferring resistance to powdery mildew are *mlo* orthologues in barley (*mlo11*; Jørgensen, 1992), tomato (*ol‐2*; Bai *et al*., 2008) and pea (*er1*; Pavan *et al*., 2011; Humphry *et al*., 2011). For these species, mutations in a single *MLO* gene are sufficient to achieve full resistance. However, in Arabidopsis, silencing of three *MLO* genes (*AtMLO2*, *AtMLO6* and *AtMLO12*) is required to obtain full resistance against powdery mildew. This indicates that, although the role of *mlo* mutation promoting resistance to different powdery mildew species is conserved, the mechanism in Arabidopsis may not be representative of the situation in other plant species. We observed that, although the Arabidopsis *edr1* mutant conferred full resistance to tomato powdery mildew, silencing of two putative tomato homologues of *EDR1* separately did not inhibit fungal sporulation (Fig. 6). There are at least two possibilities to explain this phenomenon. First, in the RNAi transformants, the tomato *EDR1* homologues still retained a low level of expression, which may be sufficient to produce enough protein for sustained functionality. This holds true especially for *Solyc01g097980*, the most likely *EDR1* orthologue of tomato based on phylogenetic analysis (Fig. 6A). Only one T2 family was obtained with the silencing construct for this gene and, although the level of silencing was significant, the expression of the gene was still relatively high (Fig. 6C). Additional tomato transformants showing a more severe reduction in expression of *Solyc01g097980* are required to elucidate the role of this gene in susceptibility towards *On*. Second, the tomato *EDR1* homologues may show redundancy, and the silencing of more than one gene may be necessary to obtain resistant plants.

In summary, we identified a natural mutation of *EDR1* in Arabidopsis accession C24‐W conferring full resistance to tomato powdery mildew *On*. We plan to investigate whether C24‐W shows resistance to additional pathogens. Furthermore, it will be of interest to reveal the overall differences between C24‐W and C24 sources not having the *edr1* mutation, which may improve our understanding of the complex resistance in C24 in general. As described for the *edr1* mutant derived from Col‐0 (Frye and Innes, 1998), our data showed that accelerated cell death and elevated *PR1* expression contribute to the resistance in the C24‐W *edr1* mutant. We plan to further study the allelic effects of the two *edr1* mutations by comparing molecular resistance mechanisms in C24‐W and Col‐0‐*edr1*.

## Experimental Procedures

### Plant growth conditions and pathogen inoculation

All the *A. thaliana* accessions were obtained from the Max Planck Institute in Köln, Germany. C24‐H was obtained from Hanzi He (Plant Physiology, Wageningen University, Wageningen, the Netherlands) and C24‐U from Dr Guido Van den Ackerveken (Plant–Microbe Interactions, Utrecht University, Utrecht, the Netherlands). The plants were grown in soil substrate in a growth chamber with a day/night cycle of 16 h/8 h at 21 °C/18 °C day/night temperature. The relative humidity was kept at 70% and the light intensity was 100 W/m^2^. The Netherlands isolate of *On* was maintained on susceptible tomato cultivar MM plants. Fungal spores were washed off from infected MM leaves with water and diluted to a concentration of 2.5 × 10^5^ spores/mL for inoculation of Arabidopsis, or 2.5 × 10^4^ spores/mL for inoculation of tomato. Approximately 30‐day‐old plants were inoculated by spraying spores on the leaves. The DI was recorded 8–14 days after inoculation of *On*: 0, no sporulation; 1, slight sporulation; 2, moderate sporulation; 3, abundant sporulation.

### QTL mapping and recombinant screening

To locate resistance loci, Joinmap 4 (Van Ooijen, 2006) and MapQTL 6 (Van Ooijen, 2009) were used with default settings. For recombinant screening, DNA was extracted using the protocol described by Kasajima *et al*. (2004). For the development of indel markers, primers were designed based on the flanking sites of known insertion and deletion polymorphisms between Col‐0 and Ler, as obtained from the Cereon database administered by Monsanto (Jander *et al*., 2002). For the development of SNP markers, the known SNPs between C24 and Col‐0 available from the 1001 genome database (http://1001genomes.org) were examined with Lightscanner™ (Idaho Technology Inc., Bioké, Leiden, Netherlands) to determine whether the SNP was applicable to distinguish C24 from Sha.

### Phylogenetic analysis of EDR1 candidates

EDR1 protein sequences from Arabidopsis Col‐0 (GenBank Accession AAG31143), *Theobroma cacao* (EOY11153), *Glycine max* (ACQ57002), *Delphinium* (BAD02482), *Oryza sativa* (AAN61142), *Hordeum vulgare* (AAG31142) and *Triticum aestivum* (AAU89661), and Solyc01g097980 and Solyc06g068980, were used as input for the phylogenetic analysis provided by Phylogeny.fr (http://www.phylogeny.fr/version2_cgi/index.cgi) (Dereeper *et al*., 2008).

### Generation of stable silenced lines

To suppress tomato genes *Solyc01g097980* and *Solyc06g068980* individually, fragments with lengths of 223 and 158 bp, respectively, were amplified from MM cDNA using primers Fw‐caccTCAGGTGCAGCGTTGGCTGAG and Rv‐TGCCCTTTGCCACATCAAGGG for *Solyc01g097980*, and primers Fw‐caccAGTGGATGGCCCCAGAAGTGCTG and Rv‐ACGGTGCTGAAACCCCACAGCG for *Solyc06g068980*. The fragments were recombined into the pENTR/D‐TOPO vector (Invitrogen Fisher Scientific, Landsmeer, Netherlands) and sequenced. Subsequently, the fragments were introduced into the pHellsgate8 vector (Helliwell *et al*., 2002) and finally transformed into *Agrobacterium* strain AGL1+virG. For the transformation of tomato cultivar MM, the same protocol as described by Huibers *et al*. (2013) was used. Primary transformants (T1) were selfed to generate T2 progeny. For each segregating T2 family, a PCR using NPTII primers (Fw‐NPTII‐TTCCCCTCGGTATCCAATTA and Rv‐NPTII‐GATTGTCTGTTGTGCCCAGT) was performed to select transgenic progeny.

### CAPS marker analysis of F_4_ progeny of C24‐W × Sha

To determine the allele composition for the *Edr1* gene in F**_4_** progeny of C24‐W × Sha, primers edr1‐S6F (TATCCACAGACTCCGCAAAG) and edr1‐S6R (TGATTCTGCGAAAACAGCAC) were designed to amplify a fragment of exon 2 and intron 2. The 526‐bp (C24‐W) or 528‐bp (Sha) PCR product was digested with *Alu*I, and the fragments were separated on an agarose gel (Fig. S2). F_4_ plants homozygous for the C24‐W allele of *edr1* showed fragments of 249, 117, 81 and 79 bp, F_4_ plants homozygous for the Sha allele of *Edr1* showed fragments of 368, 81 and 79 bp, and heterozygous F_4_ plants showed fragments of 368, 249, 117, 81 and 79 bp.

### Quantitative reverse transcription‐polymerase chain reaction (RT‐PCR) and data analysis

In each experiment, three biological replicates per genotype were used. Samples were prepared from Arabidopsis rosette leaves or pooled third and fourth leaves per tomato plant. For the quantification of fungal biomass, DNA or RNA was used. For the quantification of transcript levels, RNA was used. DNA was isolated with the DNeasy plant mini kit (Qiagen, Venlo, Netherlands). Total RNA was extracted using the RNeasy kit (Qiagen). After removal of DNA with DNase I (Invitrogen), 1 μg of total RNA was employed for cDNA synthesis using a Superscript II reverse transcriptase kit (Invitrogen). Quantitative real‐time PCR was conducted using the iQ SYBR Green Supermix (Bio‐Rad, Veenendaal, Netherlands) and the CFX96 Real‐Time system (Bio‐Rad). The PCR amplification consisted of an initial denaturation step of 3 min at 95 °C, followed by denaturation for 15 s at 95 °C, annealing and extension for 1 min at 60 °C for 39 cycles, and then a final melt step from 65 °C to 95 °C ramp with 0.5 °C increments per cycle to monitor specificity. The primers used for fungal quantification were Fw‐On‐CGCCAAAGACCTAACCAAAA and Rv‐On‐AGCCAAGAGATCCGTTGTTG. The primers used for the detection of relative transcript levels were as follows: Fw‐TGAAGGAGCCAGAAAATCCA and Rv‐TCTTCCCATGGAATCTCACA for *Solyc01g097980*; Fw‐TTCATGGGAGCTGTTACTCG and Rv‐ACTGATTGTTGGGTCGATGG for *Solyc06g068980*; Fw‐*EF*‐GGAACTTGAGAAGGAGCCTAAG and Rv‐*EF*‐CAACACCAACAGCAACAGTCT for tomato reference gene *Elongation Factor 1α* (Løvdal and Lillo, 2009); Fw‐GAACACGTGCAATGGAGTTT and Rv‐GGTTCCACCATTGTTACACCT for Arabidopsis *PR1* gene At2G14610; Fw‐CCCGTAGCATACTCCGATTT and Rv‐AAGGAGCTTAGCCTCACCAC for Arabidopsis *PR2* gene At3G57260; Fw‐AATCACAGCACTTGCACCA and Rv‐GAGGGAAGCAAGAATGGAAC for Arabidopsis reference gene actin (*act2*) At3G18780.

For the analysis of the relative expression level and fungal biomass, the 2^–ΔΔ*Ct*^ method, as described by Livak and Schmittgen (2001), was used. Data were statistically examined using independent‐samples *t*‐test and one‐way analysis of variance (ANOVA) based on *post hoc* comparisons using Tukey's honestly significant difference (HSD) test (*P* < 0.05). All analyses were performed using SPSS Statistics 20 following the instructions of the *SPSS Survival Manual*, 4th edn. (Pallant, 2010).

### Histological analyses

Three or four leaves per plant of the F_4_ progeny (C24‐W × Sha) were collected at 3, 6 and 8 dpi with *On*. At each time point, five plants homozygous for the C24‐W allele and three heterozygous plants were sampled. For 6 dpi, two homozygous plants for the Sha allele were included. The sampled leaf segments were fixed in acetic acid–ethanol (1:3, v/v) and stained with 0.03% trypan blue in lactophenol–ethanol, as described by Hering and Nicholson (1964).

## Supporting information


**Fig. S1** Relation between genotype of F_3_ progeny for quantitative trait locus‐1 (QTL‐1) and QTL‐2 and resistance to *Oidium neolycopersici*. Disease index (DI) was scored for plants showing C24 (C), heterozygous (H) or Sha (S) genotypes. (A) QTL‐1 region (both markers 159 and 162). (B) QTL‐2 region (both markers 515 and 187). Each data point represents the average value from two time points of scoring per F_3_ plant. The total number of plants with the designated genotype is shown in parentheses.

**Fig. S2** Analysis of C24‐W × Sha F_4_ plants segregating for quantitative trait locus‐1 (QTL‐1). (A) The polymorphism revealed by the CAPS marker analysis of the *EDR1* (*Enhanced Disease Resistance 1*) alleles. In total, 96 F_4_ plants were genotyped, two of which were homozygous for the Sha allele (S), 31 were homozygous for the C24‐W allele (C) and 63 were heterozygous (H). The plants were inoculated with *Oidium neolycopersici* (*On*) and scored for resistance or susceptibility. All resistant plants were homozygous for the C24‐W *edr1* allele. All heterozygous plants and the two plants homozygous for the Sha *EDR1* allele showed clear powdery mildew symptoms. (B) Images of symptoms after *On* inoculation on F_4_ plants homozygous (left) or heterozygous (right) for the C24‐W *edr1* allele.

**Fig. S3** Dinucleotide deletion in exon 2 of *EDR1* (*Enhanced Disease Resistance 1*) is specific for C24‐W. Partial sequence trace files of exon 2 of *EDR1* from C24‐W and C24‐U. The dinucleotide deletion in C24‐W is indicated. Trace files for C24‐stock and C24‐H are identical to that from C24‐U.

**Fig. S4** Application of indel markers to verify the identity of C24‐W. For each marker (see Table S5), seven plants were tested. Lane 1, Col‐0; lane 2, C24‐W; lane 3, C24‐stock; lane 4, C24‐U; lane 5, C24‐H; lane 6, C24; lane 7, Sha; M, marker. DNA from lanes 6 and 7 was employed for the development of all the markers used for mapping in this population. Genotyping was repeated twice, and data from one replicate are presented here.

**Fig. S5** Sequence of polymerase chain reaction (PCR) products containing MIR164A single nucleotide polymorphism (SNP) in different C24 sources and Col‐0.

**Fig. S6** Fungal biomass quantification in C24‐U and C24‐W compared with Col‐0 at 9 days post‐inoculation. Values were normalized relative to *act2*, and calibrated to levels in Col‐0 plants. Error bars represent standard deviation of eight biological replicates and, for each replicate, rosette leaves were collected. Asterisks indicate significant difference based on a *t*‐test: *P < 0.05; **P < 0.01.

**Table S1** Disease index (DI) scores of Arabidopsis accessions inoculated with *Oidium neolycopersici*.
**Table S2** Segregation of resistance to *Oidium neolycopersici* in Arabidopsis accessions. Chi‐squared tests were performed in all respective F_2_ generations. The *P* value is only shown when higher than 0.05, which means that the segregation ratio fits the indicated pattern.
**Table S3** Primers of indel markers for preliminary quantitative trait locus (QTL) analysis.
**Table S4** Primers of chromosome 1 markers for the genotyping of recombinants to fine map quantitative trait locus‐1 (QTL‐1).
**Table S5** Primers of indel markers for the genotyping of different sources of C24.
**Table S6** Association of plant size of F_3_ plants with markers defining the quantitative trait locus‐2 (QTL‐2) region. The F_3_ populations segregate for QTL‐2, but not for QTL‐1. They are homozygous for the Sha alleles in the QTL‐1 region. C, homozygous C‐24 allele; H, heterozygous; S, homozygous Sha allele.
