# Supplementary figures and images for: Natural loss‐of‐function mutation of EDR1 conferring resistance to tomato powdery mildew in Arabidopsis thaliana accession C24
Source: Mol Plant Pathol. 2014 Jul 9;16(1):71–82. doi: 10.1111/mpp.12165 (PMC6638503; doi:10.1111/mpp.12165)

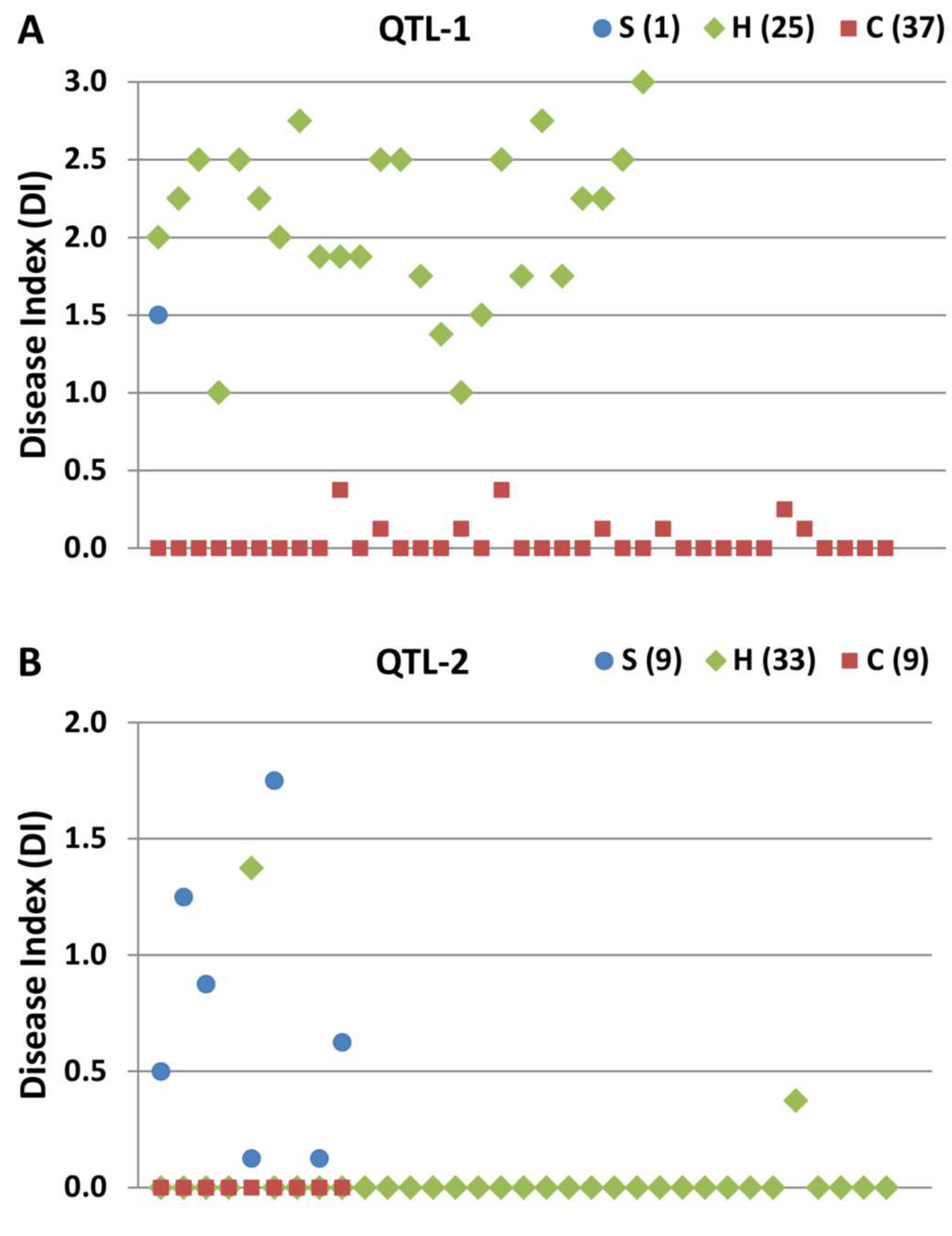

Supplement: Supplementary file 1 — Fig. S1 Relation between genotype of F3 progeny for quantitative trait locus‐1 (QTL‐1) and QTL‐2 and resistance to Oidium neolycopersici. Disease index (DI) was scored for plants showing C24 (C), heterozygous (H) or Sha (S) genotypes. (A) QTL‐1 region (both markers 159 and 162). (B) QTL‐2 region (both markers 515 and 187). Each data point represents the average value from two time points of scoring per F3 plant. The total number of plants with the designated genotype is shown in parentheses. [file MPP-16-71-s002.tiff]

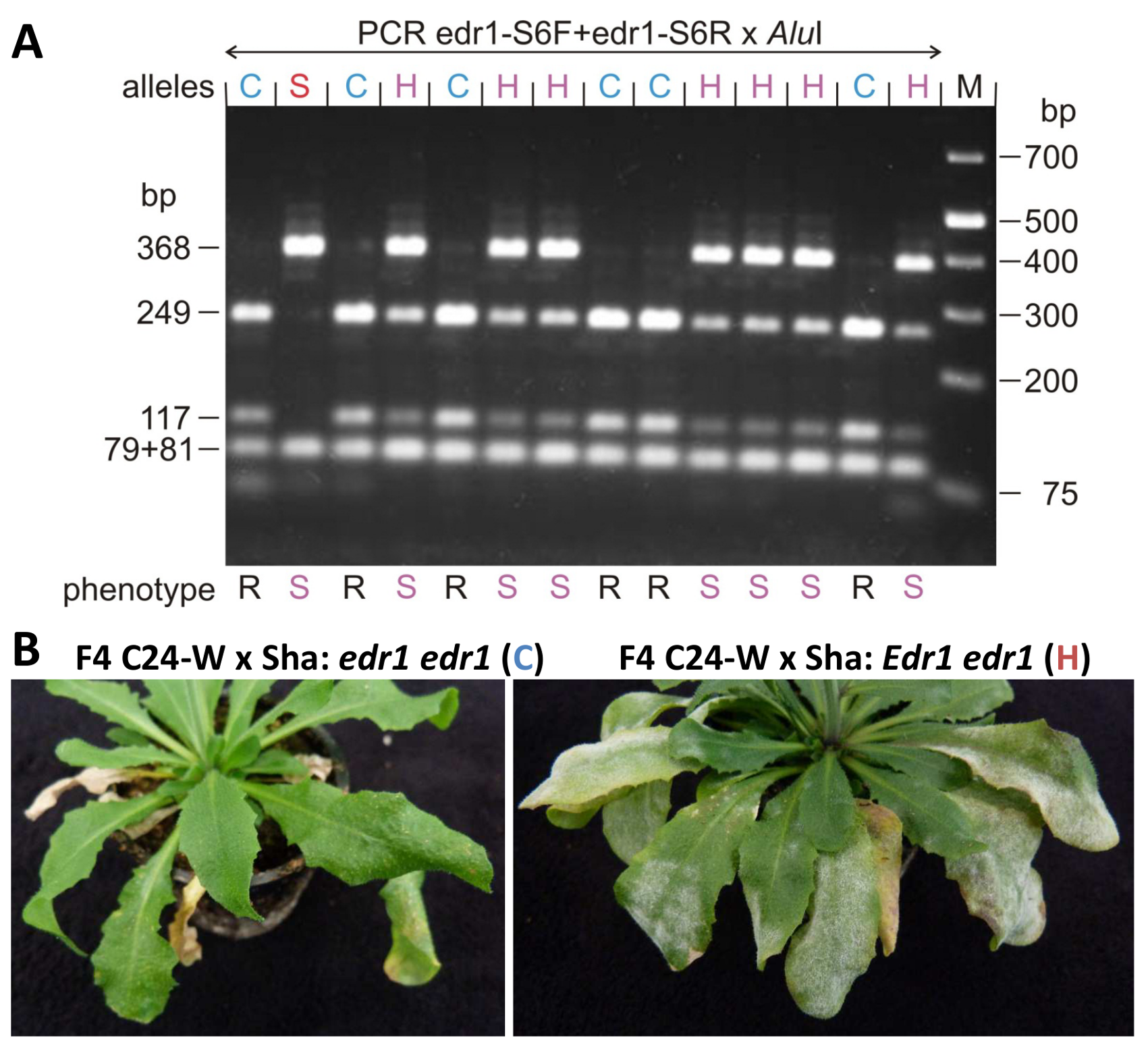

Supplement: Supplementary file 2 — Fig. S2 Analysis of C24‐W × Sha F4 plants segregating for quantitative trait locus‐1 (QTL‐1). (A) The polymorphism revealed by the CAPS marker analysis of the EDR1 (Enhanced Disease Resistance 1) alleles. In total, 96 F4 plants were genotyped, two of which were homozygous for the Sha allele (S), 31 were homozygous for the C24‐W allele (C) and 63 were heterozygous (H). The plants were inoculated with Oidium neolycopersici (On) and scored for resistance or susceptibility. All resistant plants were homozygous for the C24‐W edr1 allele. All heterozygous plants and the two plants homozygous for the Sha EDR1 allele showed clear powdery mildew symptoms. (B) Images of symptoms after On inoculation on F4 plants homozygous (left) or heterozygous (right) for the C24‐W edr1 allele. [file MPP-16-71-s003.tif]

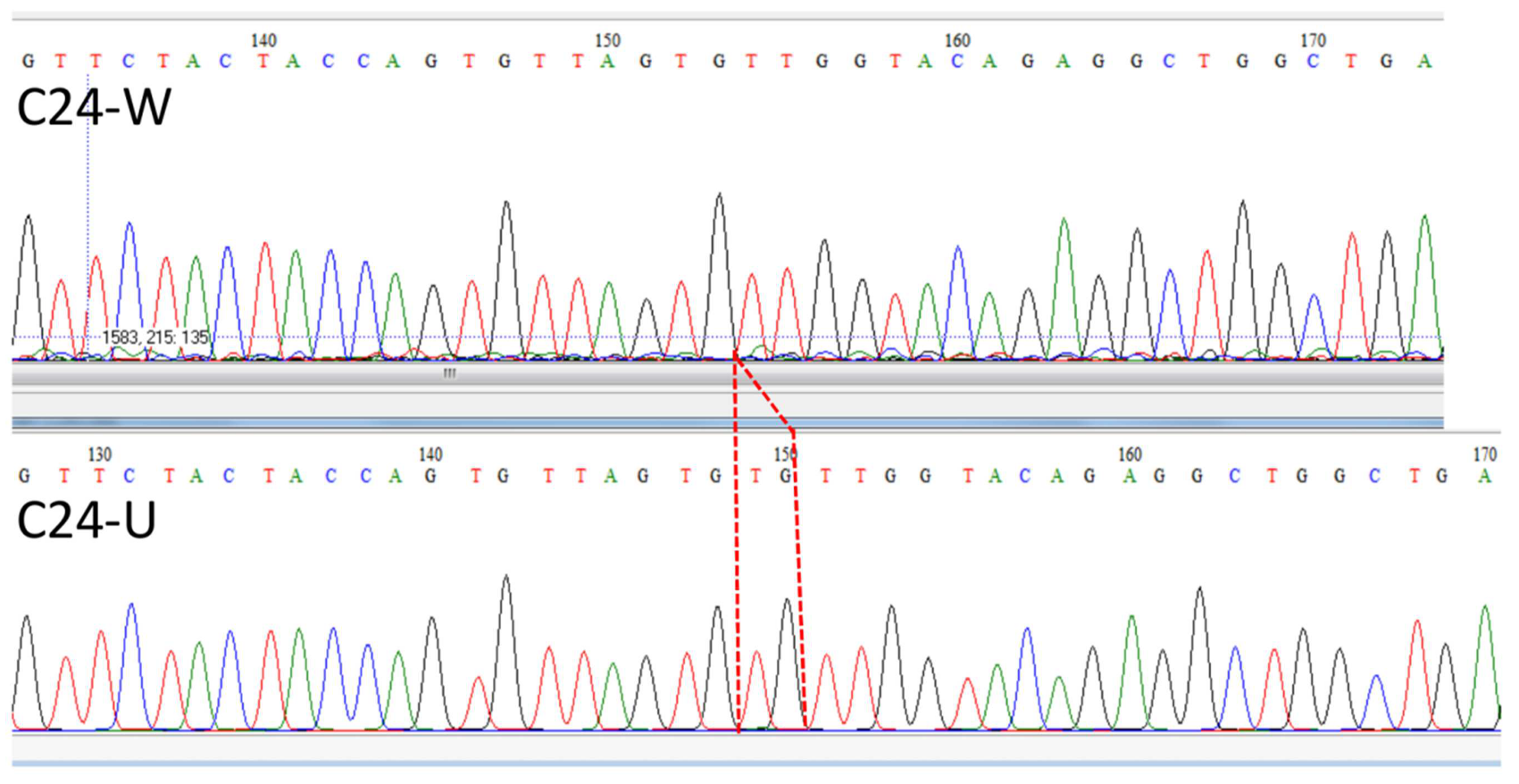

Supplement: Supplementary file 3 — Fig. S3 Dinucleotide deletion in exon 2 of EDR1 (Enhanced Disease Resistance 1) is specific for C24‐W. Partial sequence trace files of exon 2 of EDR1 from C24‐W and C24‐U. The dinucleotide deletion in C24‐W is indicated. Trace files for C24‐stock and C24‐H are identical to that from C24‐U. [file MPP-16-71-s004.tif]

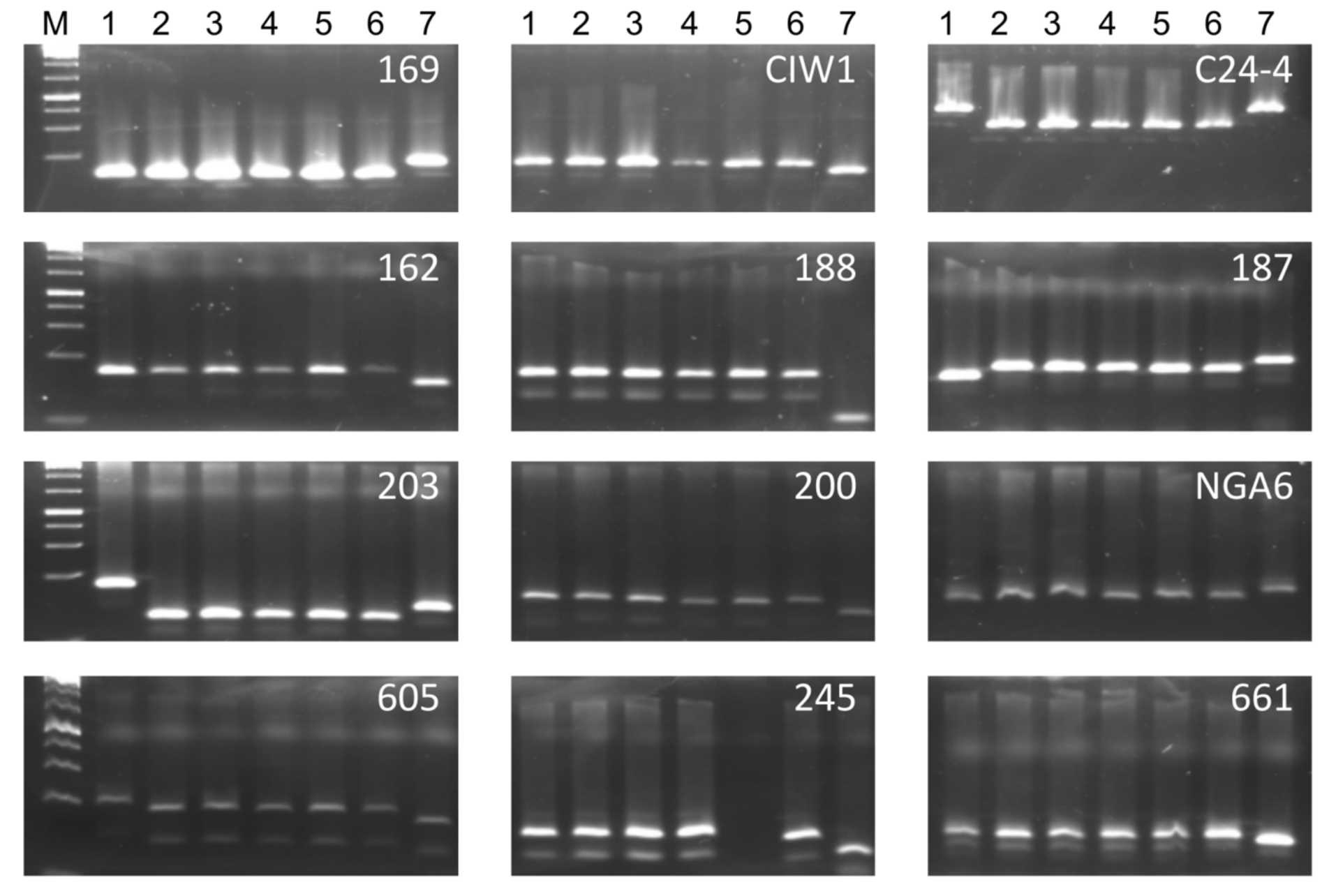

Supplement: Supplementary file 4 — Fig. S4 Application of indel markers to verify the identity of C24‐W. For each marker (see Table S5), seven plants were tested. Lane 1, Col‐0; lane 2, C24‐W; lane 3, C24‐stock; lane 4, C24‐U; lane 5, C24‐H; lane 6, C24; lane 7, Sha; M, marker. DNA from lanes 6 and 7 was employed for the development of all the markers used for mapping in this population. Genotyping was repeated twice, and data from one replicate are presented here. [file MPP-16-71-s005.tiff]

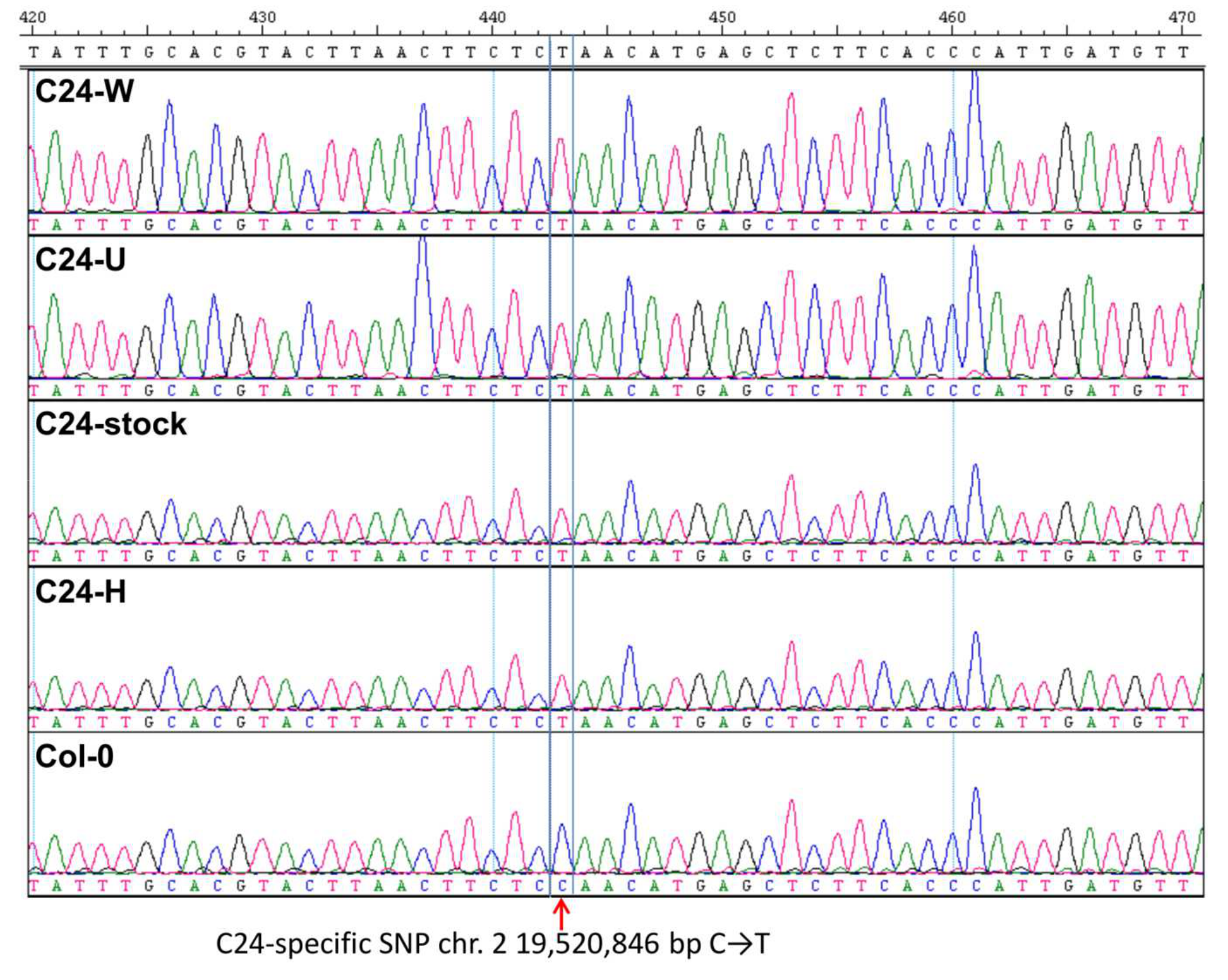

Supplement: Supplementary file 5 — Fig. S5 Sequence of polymerase chain reaction (PCR) products containing MIR164A single nucleotide polymorphism (SNP) in different C24 sources and Col‐0. [file MPP-16-71-s006.tiff]

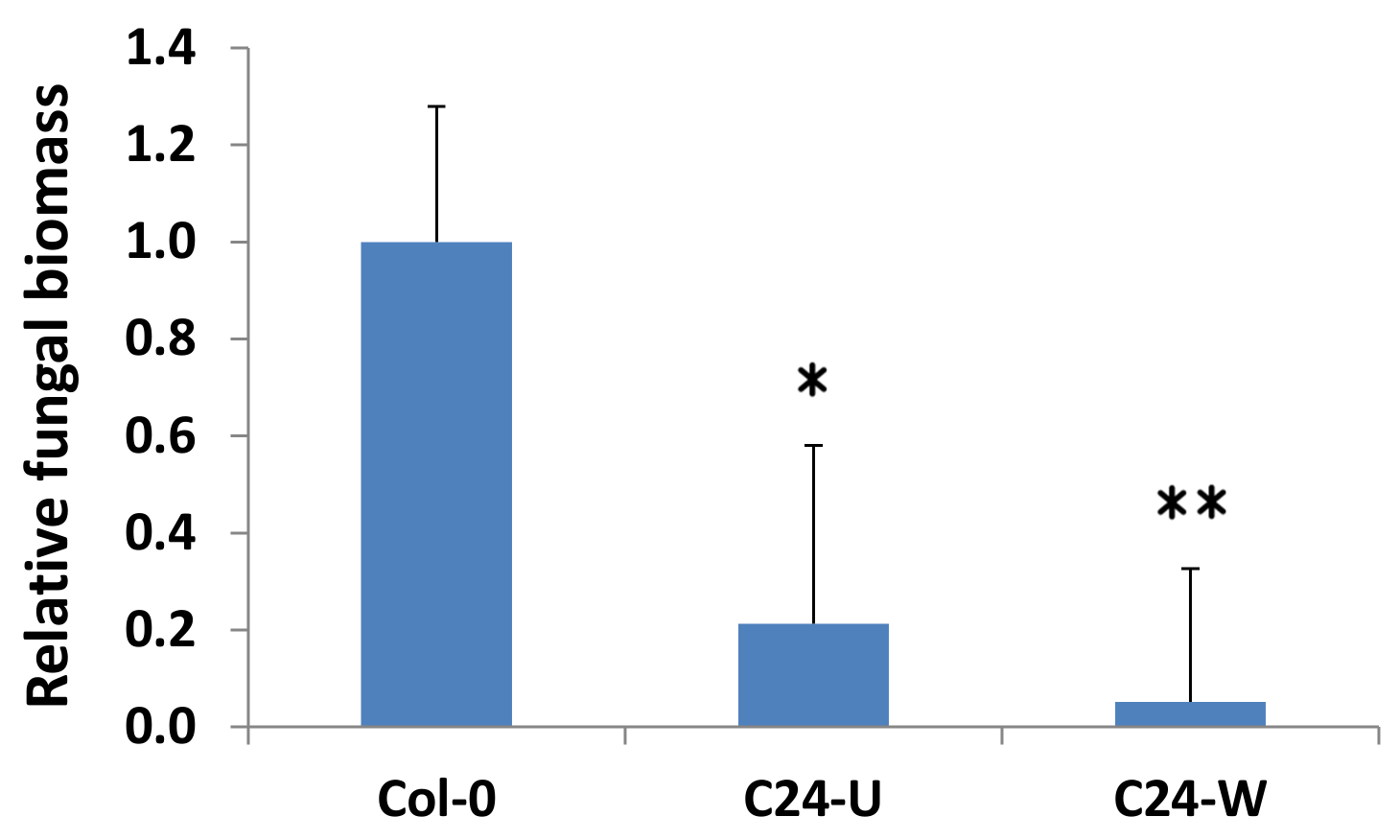

Supplement: Supplementary file 6 — Fig. S6 Fungal biomass quantification in C24‐U and C24‐W compared with Col‐0 at 9 days post‐inoculation. Values were normalized relative to act2, and calibrated to levels in Col‐0 plants. Error bars represent standard deviation of eight biological replicates and, for each replicate, rosette leaves were collected. Asterisks indicate significant difference based on a t‐test: *P < 0.05; **P < 0.01. [file MPP-16-71-s007.tif]
