# Supplementary material for: Natural loss‐of‐function mutation of EDR1 conferring resistance to tomato powdery mildew in Arabidopsis thaliana accession C24
Source: Mol Plant Pathol. 2014 Jul 9;16(1):71–82. doi: 10.1111/mpp.12165 (PMC6638503; doi:10.1111/mpp.12165)
Supplement: Supplementary file 7 — Table S1 Disease index (DI) scores of Arabidopsis accessions inoculated with Oidium neolycopersici. Table S2 Segregation of resistance to Oidium neolycopersici in Arabidopsis accessions. Chi‐squared tests were performed in all respective F2 generations. The P value is only shown when higher than 0.05, which means that the segregation ratio fits the indicated pattern. Table S3 Primers of indel markers for preliminary quantitative trait locus (QTL) analysis. Table S4 Primers of chromosome 1 markers for the genotyping of recombinants to fine map quantitative trait locus‐1 (QTL‐1). Table S5 Primers of indel markers for the genotyping of different sources of C24. Table S6 Association of plant size of F3 plants with markers defining the quantitative trait locus‐2 (QTL‐2) region. The F3 populations segregate for QTL‐2, but not for QTL‐1. They are homozygous for the Sha alleles in the QTL‐1 region. C, homozygous C‐24 allele; H, heterozygous; S, homozygous Sha allele. [file MPP-16-71-s001.doc]

**Table S1.** Disease Index (DI) scores of Arabidopsis accessions inoculated with *O. neolycopersici*.

| No. | Accession-ID | Accession | DI |  | No. | Accession-ID | Accession | DI |
| --- | --- | --- | --- | --- | --- | --- | --- | --- |
| 1 | 291 | Zal-1 | 0 |  | 63 | 6800 | Mz-0 | 0 |
| 2 | 903 | Kas-0 | 2 |  | 64 | 6810 | Nok-3 | 0 |
| 3 | 905+ | Ms-0 | 2 |  | 65 | 6818 | Ob-2 | 2 |
| **4** | **906+** | **C24** | **0** |  | 66 | 6832 | Pi-0 | 0 |
| 5 | 913 | RLD1 | 2-3 |  | 67 | 6848 | Rsch-0 | 2 |
| 6 | 925 | Litva | 0 |  | 68 | 6849 | Ri-0 | 2-3 |
| 7 | 926 | Pet-0 | 0 |  | 69 | 6865 | Stw-0 | 1-2 |
| 8 | 929 | Shah / Sha | 3 |  | 70 | 6868 | Ts-2 | 0 |
| 9 | 931 | Sorbo | 2 |  | 71 | 6884 | Van-0 | 1-2 |
| 10 | 1005 | Bsch-2 | 1-2 |  | 72 | 6885 | Wa-1 | 1 |
| 11 | 1014 | Bu-5 | 2 |  | 73 | 6918 | Te-0 | 0 |
| 12 | 1065 | Can-0 | 1 |  | 74 | 8068 | Berk | 2 |
| 13 | 1072 | Chi-0 | 0-1 |  | 75 | 8070 | Lim | 2 |
| 14 | 1074 | Chi-1 | 1 |  | 76 | 8144 | Lin | 1 |
| 15 | 1094 | CT-1 | 1 |  | 77 | 8580 | CVI | 2 |
| 16 | 1116 | Dra 0 | 2 |  | 78 | 10038 | Driel-1 | 2 |
| 17 | 1138 | En-2 | 2 |  | 79 | 10169 | Fei-0 | 0 |
| 18 | 1184 | Gd-1 | 2 |  | 80 | 10172 | Vil-0 | 2 |
| 19 | 1187 | Ge-0 | 0 |  | 81 | 10175 | FK | 1-2 |
| 20 | 1211 | Gre-0 | 1 |  | 82 | 10182 | Aa-0 | 0 |
| 21 | 1212 | GU-0 | 0 |  | 83 | 10183 | Ag-0 | 2 |
| 22 | 1214 | GU-1 | 1 |  | 84 | 10184 | Bl-1 | 0 |
| 23 | 1249 | Ji-1 | 2 |  | 85 | 10185 | Bla-10 | 2-3 |
| 24 | 1260 | Jm-1 | 2 |  | 86 | 10187 | Fl-1 | 2 |
| 25 | 1264 | Kas- 2 | 0 |  | 87 | 10189 | Pog-0 | 0 |
| 26 | 1622 | Yo-0 | 1 |  | 88 | 10210 | Hi-0 | 2 |
| 27 | 1629 | Zu-1 | 0 |  | 89 | 10212 | Pak-1 | 2 |
| 28 | 1635 | Cnt | 2 |  | 90 | 10214 | Pak-3 | 0 |
| 29 | 1636 | Nd-1 | 2 |  | 91 | 10215 | Yam-1 | 0 |
| 30 | 1637 | Ema-1 | 2 |  | 92 | 10217 | Izumo | 1-2 |
| 31 | 1639 | Wei-1 | 2 |  | 93 | 10219 | JW113 | 2 |
| 32 | 1640 | Tsu-1 | 2 |  | 94 | 10221 | TY | 1-2 |
| 33 | 1656 | Alc-0 | 2 |  | 95 | 10222 | TKS | 0 |
| 34 | 2360 | WS-2 | 3 |  | 96 | 10223 | IK | 2 |
| 35 | 6042 | Car 1 | 3 |  | 97 | 10224 | AK | 2 |
| 36 | 6044 | FLO 1 | 1-2 |  | 98 | 10225 | OY | 1 |
| 37 | 6045 | KL-PW-1 | 1 |  | 99 | 10226 | NG | 2 |
| 38 | 6047 | Mst- 1 | 2 |  | 100 | 10227 | ES | 1-2 |
| 39 | 6048 | Ken-1 | 2 |  | 101 | 10229 | Sendai-1 | 1 |
| 40 | 6182 | Wei-0 | 2 |  | 102 | 10230 | Eniwa | 0 |
| 41 | 6600 | Aa-0 | 0 |  | 103 | 10232 | RIB-1 | 2 |
| 42 | 6608 | Bay-0 | 0-1 |  | 104 | 10237 | Ost-0 (Navot) | 0 |
| 43 | 6613 | Be-0 | 3 |  | 105 | 10255 | Kam (Navot) | 0 |
| 44 | 6621 | Bla-6 | 0 |  | 106 | 10256 | Strand | 2 |
| 45 | 6626 | Br-0 | 2 |  | 107 | 10257 | Byn | 0 |
| 46 | 6627 | Bs-1 | 2 |  | 108 | 10258 | Orn | 0 |
| 47 | 6643 | Bur-0 | 0 |  | 109 | 10265 | Pont L'Eveqeu | 0 |
| 48 | 6645 | Blh-1 | 1-2 |  | 110 | 10270 | Son Stefano | 2 |
| 49 | 6659 | Cal-0 | 0 |  | 111 | 10281 | Sij-1 | 1-2 |
| 50 | 6669 | Co-1 | 0 |  | 112 | 10282 | Sij-2 | 2 |
| 51 | 6672 | Co-4 | 1-2 |  | 113 | 10296 | Daejeon | 0 |
| 52 | 6689 | Ei-2 | 0 |  | 114 | 10297 | Suwon | 0 |
| 53 | 6693 | Eil-0 | 2 |  | 115 | 22351 | HS-1 | 2 |
| 54 | 6699 | Es-0 | 0 |  | 116 | 22363 | Ith-1 | 0 |
| 55 | 6700 | Est-0 | 0 |  | 117 | 22401 | Kno-1 | 1 |
| 56 | 6705 | Fi-1 | 1 |  | 118 | 22419 | Csh-1 | 2 |
| 57 | 6714 | Ga-0 | 0 |  | 119 | 22436 | KZ-2 | 1-2 |
| 58 | 6732 | Gy-0 | 0 |  | 120 | 22445 | KZ-13 | 2 |
| 59 | 6752 | Ka-0 | 1 |  | 121 | 22446 | Puz-2 | 0 |
| 60 | 6754 | Kil-0 | 2 |  | 122 | 22456 | Sapporo | 2 |
| 61 | 6755 | Kin-0 | 1-2 |  | 123 | 22491 | Konchezero | 1-2 |
| 62 | 6799 | Mt-0 | 0-1 |  |  | | | |

**Table S2.** Segregation of resistance to *Oidium neolycopersici* in Arabidopsis accessions. Chi-square tests were performed in all respective F_2_ generations. The P value is only shown when higher than 0.05, which means that the segregation ratio fits the indicated pattern.

|  |  | F_2_ (R:S) ^1^ | Segregation 3 : 1 | Segregation 1 : 3 | F_2_  (R:I:S) ^2^ | Segregation 1 : 2 : 1 |
| --- | --- | --- | --- | --- | --- | --- |
| Cross | F_1_ phenotype^1^ |  | *P (*χ^2^ test) | *P* (χ^2^ test) |  | *P* (χ^2^ test) |
| Bla-6 (♀) x Col-0 (♂) | R | (72:20) | *P*=0.470 |  | (72:7:13) |  |
| Bay-0 (♀) x Sha (♂) | S | (22:72) |  | *P*=0.721 | (22:55:17) | *P*=0.196 |
| Kas-2 (♀) x Col-0 (♂) | S | (17:66) |  | *P*=0.342 | (17:33:33) |  |
| Litva (♀) x Col-0 (♂) | S | (17:79) |  | *P*=0.099 | (17:51:28) | *P*=0.235 |
| Es-0 (♀) x Ws-2 (♂) | S | (14:78) |  |  | (14:32:46) |  |
| Zal-1 (♀) x Col-0 (♂) | S | (10:62) |  |  | (10:33:29) |  |
| Pak-3 (♀) x Sha (♂) | S | (38:56) |  |  | (38:50:6) |  |
| **C24 (♀) x Sha (♂)** | **S** | **(53:43)** |  |  | **(53:30:13)** |  |
| GU-0 (♀) x Sha (♂) | S | (52:38) |  |  | (52:37:1) |  |
| Cal-0 (♀) x Col-0 (♂) | S | (49:43) |  |  | (49:37:6) |  |
| Ga-0 (♀) x Sha (♂) | S | (53:34) |  |  | (53:34:0) |  |
| Nok-3 (♀) x Col-0 (♂) | S | (6:83) |  |  | (6:78:5) |  |
| Pi-0 (♂) x Col-0 (♀) | S | (87:5) |  |  | (87:4:1) |  |
| Te-0 (♀) x Sha (♂) | S | (38:58) |  |  | (38:53:5) |  |
| Fei-0 (♂) x Col-0 (♀) | S | (12:83) |  |  | (12:73:10) |  |
| Bl-1 (♀) x Col-0 (♂) | S | (81:13) |  |  | (81:13:0) |  |
| TKS (♀) x Col-0 (♂) | S | (44:51) |  |  | (44:42:9) |  |
| Eniwa (♀) x Col-0 (♂) | S | (95:5) |  |  | (95:4:1) |  |
| 10265 (♀) x Col-0 (♂) | S | (38:52) |  |  | (38:45:7) |  |

^1^ Plants with DI=0 were scored as resistant (R), while plants with DI>0 were scored as susceptible (S).

^2^ Plants with DI=0 were scored as R, with 0<DI<2.5 as intermediate (I), with DI=2.5-3 as S.

**Table S3.** Primers of Indel markers for preliminary QTL analysis

| Indel | Chromosome | Forward | Reverse |
| --- | --- | --- | --- |
| 159 | 1 | GATGAATTCTTCCTTTTCACGTT | TGTTGTACTTAAATGTAACCAGTCAG |
| F21M12 | 1 | GGCTTTCTCGAAATCTGTCC | TTACTTTTTGCCTCTTGTCATTG |
| 162 | 1 | CATACATACAATTCACTAACCAAAA | TGGATCTCCTTAATAGTTTAAAAGG |
| 561 | 1 | GGACAACGTCTCAAACGGTT | GGAGGCTATACGAATCTTGACA |
| CIW1 | 1 | ACATTTTCTCAATCCTTACTC | GAGAGCTTCTTTATTTGTGAT |
| 567 | 1 | CAACCACCAGGCTC | GTCAAACCAGTTCAATCA |
| 169 | 1 | CAGAATTTCTATCTGAAGAATCGAG | GTGAAGGTTTAGAGAGAATCAAAGG |
| 515 | 2 | ATCTTCCTCCGACGACATTG | TGATGATATGTTTCCCCTCGT |
| 187 | 2 | GAGAAATATCGACGGGAAAAA | ATGCTCATCTTACAACAACACTAAA |
| 188 | 2 | AAAGAGTCAAGGAAAAGTATGTGTG | TTAAGATAGAAACCAAAACCAAGC |
| 189 | 2 | GTTTGCGTTTAATAGTCAAGATATG | CAAATGTTTAAGGTTTGTGGTTG |
| 585 | 3 | AGCTGCTTCCTTATAGCGTCC | CATCCGAATGCCATTGTTC |
| 200 | 3 | AAATAAGATTTGTAATGTAAGACGAA | TTCCACCTAACCTAATAATAACAAG |
| 591 | 3 | GCACTTGCAGCTTAACTT | CGTGACTGTCAAACCG |
| 203 | 3 | GAACAATAAAGAGGAAGAAGAAAGC | GCATTACAACGTATAACGTAATGAAA |
| 661 | 3 | ATGGATGCATTTGGAAGAAA | TTGTGTAATTGATTTTACGTCATTTT |
| NGA6 | 3 | TGGATTTCTTCCTCTCTTCAC | ATGGAGAAGCTTACACTGATC |
| 218 | 4 | GACATAACTTCGAATTGTTGGATAG | AATTTCGCCGGAATAAACAG |
| 605 | 4 | TTTCTTGTCTTTCCCCTGAA | GACGAAGAAGGAGACGAAAA |
| 611 | 4 | CGTTTCATCAAGTTCCGA | TAGGAGGTTATCATGCGTG |
| 245 | 5 | GCAATATCAGGGTCTTGTAAAGATA | CCATTGGATATAATTAAGAAGAAGAA |

**Table S4.** Primers of chromosome 1 markers for genotyping recombinants to fine-map QTL-1

| Marker | Type | Chromosome location | Forward | Reverse |
| --- | --- | --- | --- | --- |
| 159 | Indel | 440401 | GATGAATTCTTCCTTTTCACGTT | TGTTGTACTTAAATGTAACCAGTCAG |
| 30 | Indel | 2579701 | CTCTTGGTGGTGTCCCAAGT | TCGACGCAGTTTTTCATCAG |
| SNP1 | SNP | 2754401 | GCCGTGGATCAAACCCTTAT | TGCATTCATGAAAGGGGAAT |
| SNP23 | SNP | 2801501 | TGTTGTCGATTGGCTGAGAA | AATGGTAGCCGCAGCAATAG |
| SNP50 | SNP | 2811983 | ATAAACGTGCCTGCGATTTC | GTGGTTCCAATGGCATCTTT |
| SNP54 | SNP | 2825828 | TCTGGTGATTGAAGAGAACCTC | TTCTTGCAGGGCCTCTATTG |
| SNP46 | SNP | 2829144 | CATTGGTCTCCAGGGCTAAA | GCTTTGAGCCACACTAAGCTC |
| 73 | Indel | 2890801 | TTGTGGATTATGAAGGAAAAACA | CGGCACAAAAGTGTTAACGAG |
| F21M12 | Indel | 3212189 | GGCTTTCTCGAAATCTGTCC | TTACTTTTTGCCTCTTGTCATTG |
| 38 | Indel | 3319641 | TTGCCAATTATAGGTTGACACG | TTCAATTGTTGCCACGCATA |
| 162 | Indel | 8103701 | CATACATACAATTCACTAACCAAAA | TGGATCTCCTTAATAGTTTAAAAGG |

**Table S5.** Primers of Indel markers for genotyping different sources of C24

| Indel | Chromosome | Forward | Reverse |
| --- | --- | --- | --- |
| 169 | 1 | CAGAATTTCTATCTGAAGAATCGAG | GTGAAGGTTTAGAGAGAATCAAAGG |
| CIW1 | 1 | ACATTTTCTCAATCCTTACTC | GAGAGCTTCTTTATTTGTGAT |
| C24-4 | 1 | AAGCCAAGTACCTCCAAGCA | TTTCCCTCAAGGGTTCTTCA |
| 162 | 1 | CATACATACAATTCACTAACCAAAA | TGGATCTCCTTAATAGTTTAAAAGG |
| 188 | 2 | AAAGAGTCAAGGAAAAGTATGTGTG | TTAAGATAGAAACCAAAACCAAGC |
| 187 | 2 | GAGAAATATCGACGGGAAAAA | ATGCTCATCTTACAACAACACTAAA |
| 203 | 3 | GAACAATAAAGAGGAAGAAGAAAGC | GCATTACAACGTATAACGTAATGAAA |
| 200 | 3 | AAATAAGATTTGTAATGTAAGACGAA | TTCCACCTAACCTAATAATAACAAG |
| NGA6 | 3 | TGGATTTCTTCCTCTCTTCAC | ATGGAGAAGCTTACACTGATC |
| 605 | 4 | TTTCTTGTCTTTCCCCTGAA | GACGAAGAAGGAGACGAAAA |
| 661 | 4 | ATGGATGCATTTGGAAGAAA | TTGTGTAATTGATTTTACGTCATTTT |
| 245 | 5 | GCAATATCAGGGTCTTGTAAAGATA | CCATTGGATATAATTAAGAAGAAGAA |

**Table S6.** Association of plant size of F3 plants with markers defining the QTL-2 region. The F3 populations segregate for QTL-2 but not for QTL-1. They are homozygous for the Sha alleles in the QTL-1 region. C, homozygous C-24 allele; H, heterozygous; S, homozygous Sha allele.

| Plant  size | QTL-2 marker 515 | | | QTL-2 marker 187 | | |
| --- | --- | --- | --- | --- | --- | --- |
|  | C | H | S | C | H | S |
| Small | 10 | 11 | 2 | 9 | 14 | 1 |
| Medium | 6 | 37 | 1 | 12 | 23 | 9 |
| Big | 0 | 6 | 18 | 1 | 12 | 11 |
